# Identification of ULK1 as a novel biomarker involved in miR-4487 and miR-595 regulation in neuroblastoma SH-SY5Y cell autophagy

**DOI:** 10.1038/srep11035

**Published:** 2015-07-17

**Authors:** Yi Chen, Shuya Wang, Lan Zhang, Tao Xie, Sicheng Song, Jian Huang, Yonghui Zhang, Liang Ouyang, Bo Liu

**Affiliations:** 1 Department of Gastrointestinal Surgery, State Key Laboratory of Biotherapy, Collaborative Innovation Center of Biotherapy, West China Hospital, Sichuan University, Chengdu 610041, China; 2 Northwestern University, Feinberg School of Medicine, 303 East Chicago Avenue, Chicago, Illinois 60611, USA; 3 School of Traditional Chinese Materia Medica, Shenyang Pharmaceutical University, Shenyang, 110016, China; 4 Collaborative Innovation Center for Biotherapy, Department of Pharmacology & Pharmaceutical Sciences, School of Medicine, Tsinghua University, Beijing 100084, China

## Abstract

Autophagy, referring to an evolutionarily conserved, multi-step lysosomal degradation process, has been well-known to be initiated by Unc-51 like kinase 1 (ULK1) with some links to Parkinson’s disease (PD). MicroRNAs (miRNAs), small and non-coding endogenous RNAs 22 ~ 24 nucleotides (nt) in length, have been demonstrated to play an essential role for modulating autophagy. Recently, the relationships between miRNAs and autophagy have been widely reported in PD; however, how microRNAs regulate autophagy still remains in its infancy. Thus, in this study, we computationally constructed the ULK1-regulated autophagic kinase subnetwork in PD and further identified ULK1 able to negatively regulate p70^S6K^ in starvation-induced autophagy of neuroblastoma SH-SY5Y cells. Combination of *in silico* prediction and microarray analyses, we identified that miR-4487 and miR-595 could target ULK1 and experimentally verified they could negatively or positively regulate ULK1-mediated autophagy. In conclusion, these results may uncover the novel ULK1-p70^S6K^ autophagic pathway, as well as miR-4487 and miR-595 as new ULK1 target miRNAs. Thus, these findings would provide a clue to explore ULK1 and its target miRNAs as potential biomarkers in the future PD therapy.

MicroRNAs (miRNAs), small and non-coding RNA molecules of 22 ~ 24 nucleotides in length, have been well-known to regulate functions of the gene expression at both transcriptional and post-transcriptional levels^1^. Hitherto, about 2,000 miRNA genes have been found since small non-coding RNAs including lin-4 and let-7 were identified in *Caenorhabditis* elegans (*C. elegans*)^2^. The first miRNA lin-4 was discovered in the year of 1993, when a small RNA encoded by the lin-4 locus was associated to the developmental timing of the nematode by modulating the protein lin-14, but it was thought to be an idiosyncrasy at that time^3^. Only after let-7 was characterized and identified to be conserved in many model organisms, the importance of miRNAs in physiology and pathology are beginning to emerge^4^. Since then, miRNAs have been found to be involved in a series of homeostatic processes, including cell survival, proliferation, apoptosis and autophagy^5^,^6^.

Of note, autophagy, a term from Greek “auto” (self) and “phagy” (to eat), refers to an evolutionarily conserved, multi-step lysosomal degradation process in which a cell degrades long-lived proteins and damaged organelles^7^. Three forms of autophagy have been identified, among which macroautophagy (hereafter referred to autophagy) is the major regulated mechanism^8^. It is initiated by the formation of double-membrane-bound vesicles namely autophagosomes and is highly regulated by a number of autophagy-related genes (Atgs) such as ULK1, Atg5, Beclin-1 and LC3, which can play a crucial role in autophagosome formation and autophagy regulation^9^,^10^. ULK1, an Unc-51 like kinase1, is crucial in the initiation of the autophagy process^11^.

Misfolded protein is considered as one of the major events to activate a defense mechanism and may lead to programmed cell death in multiple neurodegenerative diseases, including Parkinson’s disease (PD). PD is a progressive neurodegenerative disease characterized by the accumulation of Lewy bodies and by loss of nigrostriatal dopaminergic neurons^12^. Thus, abnormal dopaminergic system and protein disposal failure can be integrated into few PD cellular models, such as SH-SY5Y cells. SH-SY5Y cell is a kind of human neuroblastoma cells possessing a complete dopaminergic system, which can particularly couple the good activity of the dopamine transporter with the low activity of the vesicular monoamine transporter type II^13^. Administration of exogenous dopamine in the SH-SY5Y culture medium may raise the cytoplasmic dopamine concentration, leading to apoptotic cell death; thus, SH-SY5Y cell can be developed as a cellular model for PD investigation, which has been verified by plentiful researches^14^.

Recently, some studies have reported that autophagy, as a conserved homeostatic process for degrading long-lived proteins and damage organelles, may contribute to neurodegeneration in PD^15^,^16^. In addition, other studies have also reported that miRNAs are pathologically altered during the inexorable course of PD, suggesting that miRNAs may be the contributing factors in PD^17^,^18^. As mentioned above, bridging the gap between miRNAs and autophagy would be impressive for better understanding of their intricate relationships and thus providing more novel therapeutic strategies in PD. Based upon *in silico* analysis and experimental validation, we found that ULK1 could inhibit p70^S6K^ in starvation-induced autophagy, and further identified that miR-4487 and miR-595 were novel ULK1 target miRNAs. These findings would shed light on elucidating ULK1 and its target microRNAs as potential new biomarkers for future PD therapy.

## Results

### Autophagic kinase subnetwork in neurodegenerative disease and starvation- induced SH-SY5Y cell autophagy

**Figure 1:**
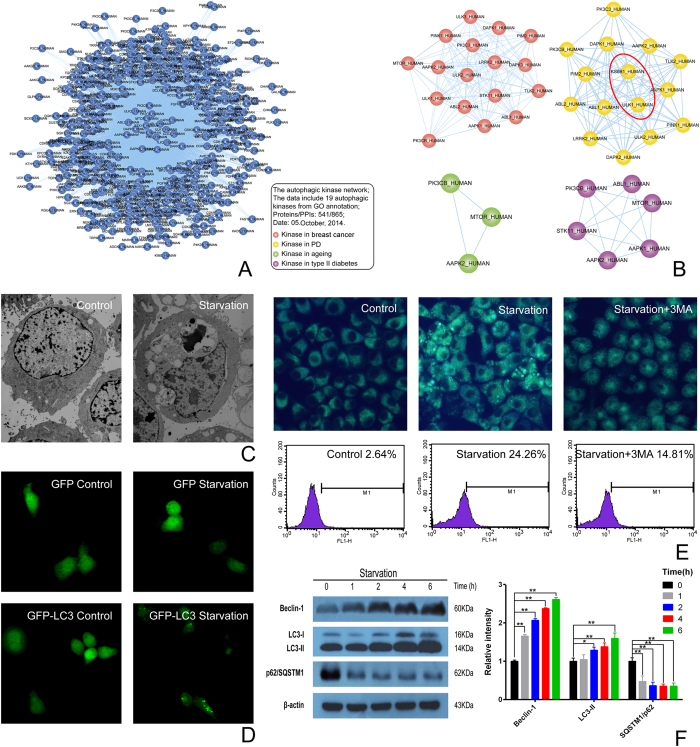
The ULK1 subnetwork of Parkinson’s disease and starvation-induced SH-SY5Y cell autophagy (**A**) The autophagic kinase network; (**B**) Dynamic kinase-regulated autophagic subnetwork in different diseases: (a) Breast cancer; (b) Parkinson’s disease; (c) Ageing; (d) Type II diabetes; (**C**) These characteristic changes were observed by transmission electron microscopy; (**D**) The formation of autophagic vacuoles was assessed by GFP-LC3 distribution; (**E**) The morphologic changes were observed under a fluorescence microscope by MDC staining; (**F**) The expression of Beclin-1 was upregulated and the ratio of LC3-II/LC3-I was also increased. And, the expression of p62/SQSTM1 was downregulated. The SH-SY5Y cells were treated for 0, 1, 2, 4, 6 h by starvation, respectively. Then, whole cell lysates were subjected to Western blots, β-actin was used as a loading control, the data are representative of 3 independent experiments. ^*^P < 0.05; ^**^P < 0.01.

Based upon the data from PrePPI, we constructed the autophagic kinase network, which was composed of 541 proteins and 865 PPIs (Fig. 1A). Then, we refined the autophagic kinase network into different disease-related subnetworks by using different types of autophagic gene microarray, including breast cancer, PD, ageing and type II diabetes, respectively (Fig. S1). Interestingly, we found that ULK1 (ULK1_HUMAN) plays a key role in interacting with other kinases, such as p70^S6K^ (KS6B1_HUMAN), in the autophagic kinase subnetwork of PD (Fig. 1B).

Typical characteristics of starvation-induced autophagy were observed in SH-SY5Y cells under starvation. These characteristic changes included extensive cytoplasm vacuolization, and these autophagic vacuoles contained degraded organelles by transmission electron microscopy (Fig. 1C). The formation of autophagic vacuoles was assessed by GFP-LC3 distribution. Control cells presented diffused staining, and ULK1 activation resulted in extensive GFP-LC3 localization (Fig. 1D). The morphologic changes of autophagy were also observed under a fluorescence microscope by MDC staining (Fig. 1E). To further investigate the role of autophagy in SH-SY5Y cells, we examined the expressions of Beclin-1, LC3 and p62/SQSTM1. The expression of Beclin-1 was upregulated and the ratio of LC3-II/LC3-I was also increased. In addition, the expression of p62/SQSTM1 was decreased (Fig. 1F). These observations indicate that starvation induces autophagy in SH-SY5Y cells.

### ULK1 negatively regulates p70^S6K^ in SH-SY5Y cell autophagy

**Figure 2:**
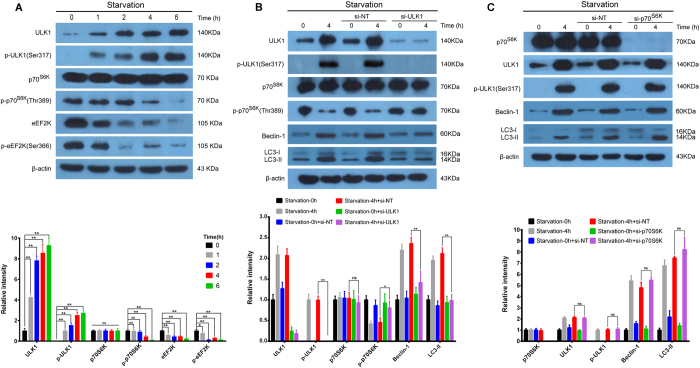
ULK1 negatively regulates p70^S6K^ in SH-SY5Y cell autophagy (**A**) When the expression of p-ULK1 was increased, the expressions of p-p70^S6K^ and eEF2K were decreased. The SH-SY5Y cells were treated for 0, 1, 2, 4, 6 h by starvation, respectively. Then, whole cell lysates were subjected to Western blots, β-actin was used as a loading control, the data are representative of 3 independent experiments. ^*^P < 0.05; ^**^P < 0.01. (**B**) Using siRNA-ULK1 in SH-SY5Y cell autophagy, the expression of p-p70^S6K^ was increased, and using siRNA-p70^S6K^ in SH-SY5Y cell autophagy, the expression of p-ULK1 was not changed compared to the negative control group. The SH-SY5Y cells were transfected with siRNAs for 48 h, followed by starvation for 0 or 4 h, then subjected to immunoblot, β-actin was used as a loading control, the data are representative of 3 independent experiments. ^*^P < 0.05; ^**^P < 0.01.

Based upon our aforementioned prediction that ULK1 could interact with p70^S6K^, we found that when the expressions of ULK1 and p-ULK1 were increased, the expressions of p-p70^S6K^ and eEF2K were decreased, suggesting that ULK1 may inhibit p70^S6K^ and eEF2K in SH-SY5Y cell autophagy (Fig. 2A). To extend our study of ULK1 with p70^S6K^ and eEF2K, we used siRNA-ULK1 in SH-SY5Y cell autophagy, and found that the expression of p-p70^S6K^ was increased comparing to negative controls, while the ratio of LC3-II/LC3-I was decreased, suggesting ULK1 may negatively regulate p70^S6K^ in SH-SY5Y cell autophagy (Fig. 2B). To evaluate the role of mTOR in ULK1 regulating p70^S6K^, rapamycin, an inhibitor of mTOR, was applied as a positive control mimicking starvation condition. We found that expressions of p-mTOR, p-p70^S6K^ and p-4E-BP1 were decreased by starvation or rapamycin treatments. The siRNA-ULK1 treatment could significantly restore the inhibition of mTOR, p70^S6K^ and 4E-BP1, indicating that ULK1 may regulate p70^S6K^ via mTOR (Fig. S2). On the other hand, we used siRNA-p70^S6K^ in SH-SY5Y cell autophagy and found that the expression of p-ULK1 was not changed compared to the negative control group, suggesting p70^S6K^ may not affect ULK1 in SH-SY5Y cell autophagy (Fig. 2C). Therefore, these results suggest that ULK1 negatively regulates p70^S6K^ in SH-SY5Y cell autophagy.

### *In silico* prediction and microarray-based identification of ULK1 target miRNAs in autophagy

**Figure 3:**
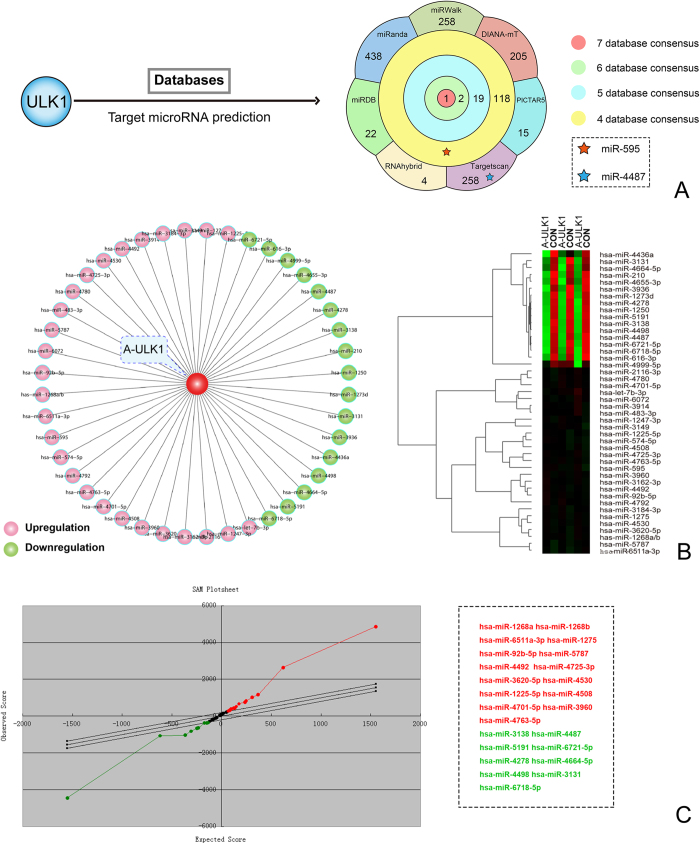
*In silico* prediction and microarray-based identification of potential target miRNAs of ULK1 in autophagy (**A**) Target microRNA prediction of ULK1; (**B**) In microarray analysis, 27 microRNAs were upregulated whereas 17 microRNAs were downregulated in SH-SY5Y cell autophagy; (**C**) Based upon SAM analysis, there are 24 differentially expressed miRNAs. 15 of them were significantly up-regulated and 9 were significantly down-regulated.

On the basis of the ULK1 subnetwork in PD, we used a series of target microRNA prediction software to identify potential target microRNAs of ULK1. We found that 258 miRNAs were predicted by miRWalk; 205 by DIANA-mT; 15 by PICTAR5; 258 by TargetScan; 4 by RNAhybrid; 22 by miRDB and 438 by miRanda. Amongst them, 118 miRNAs were predicted by 4 databases. 19 of them were from 5 databases. Only 2 and 1 miRNAs were predicted by 6 and 7 databases, respectively. Notably, miR-595 was predicted by 5 databases, and miR-4487 was only predicted by TargetScan. Afterwards, the two miRNAs were further evidenced as target microRNAs of ULK1 (Fig. 3A).

Next, we designed and made the microRNA profiling between the control SH-SY5Y cells and the starvation-induced autophagic SH-SY5Y cells. In microRNA profiling, we found that 27 microRNAs were upregulated whereas 17 microRNAs were downregulated in SH-SY5Y cell autophagy (Fig. 3B). In this study, we found 24 differentially expressed miRNAs: 15 of them were significantly up-regulated and 9 were significantly down-regulated (Fig. 3C). As the result of significance analysis of microarrays (SAM) analysis, miR-4487 was significantly down-regulated, was selected for further experiment. Combined with the above-mentioned target microRNA prediction, miR-595, as four database consensus result, was up-regulated. Thus, these results indicated that miR-4487 and miR-595 may be identified as potential target miRNAs of ULK1 in SH-SY5Y cell autophagy, and we carried out experiments to identify the divergent expression microRNAs between the control SH-SY5Y cell and starvation-induced autophagy of SH-SY5Y cells.

### MiR-4487 and miR-595 are identified to negatively or positively regulate ULK1 in SH-SY5Y cell autophagy, respectively

**Figure 4:**
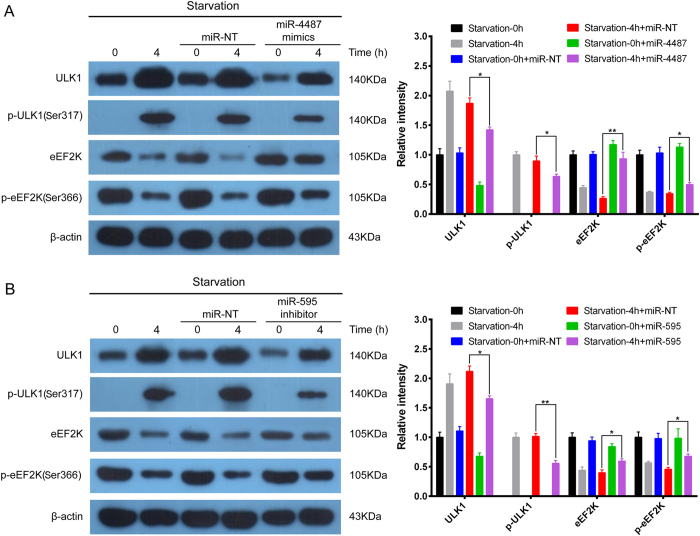
miR-4487 and miR-595 regulate ULK1-mediated autophagic pathway in SH-SY5Y cells (**A**) Under the condition of starvation-induced autophagy, miR-4487 mimetic could remarkably decrease the expressions of ULK1 but increased p-eEF2K (ser366) expressions. The SH-SY5Y cells were transfected with miR-4487 mimetic for 48 h, followed by starvation for 0 or 4 h, then subjected to Western blots, β-actin was used as a loading control, the data are representative of 3 independent experiments. ^*^P < 0.05; ^**^P < 0.01. (**B**) miR-595 inhibitor could decrease the expressions of ULK1 but increased p-eEF2K (ser366) expressions at some level. The SH-SY5Y cells were transfected with miR-595 inhibitor for 48 h, followed by starvation for 0 or 4 h, then subjected to Western blots, β-actin was used as a loading control, the data are representative of 3 independent experiments. ^*^P < 0.05; ^**^P < 0.01.

Besides being ULK1 target miRNAs, miR-4487 and miR-595 were also predicted to target eEF2K, which is a downstream kinase of p70^S6K^. Thus, under the condition of starvation-induced autophagy, we found that miR-4487 mimetic could remarkably decrease the expression of ULK1 but increased p-eEF2K (ser366) expression at some level, suggesting that miR-4487 negatively regulates ULK1 and thus inhibiting autophagy in SH-SY5Y cells (Fig. 4A). In addition, we found that miR-595 inhibitor could remarkably decrease the expression of ULK1 but increased p-eEF2K (ser366) expression at some level, suggesting that miR-595 positively regulates ULK1 and thus inducing autophagy in SH-SY5Y cells (Fig. 4B). These results indicate that miR-4487 and miR-595 may negatively or positively regulate ULK1-mediated autophagic pathway in SH-SY5Y cells, respectively.

## Discussion

In the autophagy process, Inhibition of mTOR by starvation or rapamycin leads to dephosphorylation of ULK1 and mAtg13 and activating ULK1 to phosphorylate FIP200, thus inducing autophagy^19^. Moreover, p70^S6K^ phosphorylates eukaryotic elongation factor 2 kinase (eEF2K) and thus relieving elongation factor 2 (eEF2) from the negative regulation of eEF2K as well as inhibiting/inducing autophagy. They are well-known to be the two downstream pathways of mTORC1 in autophagy^20^. Thus, ULK1 seems to have no relationship with p70^S6K^ or eEF2K in autophagy. Recently, silencing of eEF2K has been reported to promote autophagic survival via activation of the AMPK-ULK1 pathway in colon cancer cells, suggesting eEF2K can regulate ULK1 indirectly^21^. However, in our study, we found that ULK1 activation could inhibit the expression of p-p70^S6K^ or p-eEF2K. And, we found that si-p70^S6K^ had no effect on ULK1 but si-ULK1could decrease p-p70^S6K^ expression, suggesting that ULK1 may negatively regulate p70^S6K^ in SH-SY5Y cell autophagy.

Next, we combined target microRNA prediction with microarray analyses to confirm potential microRNAs that can target ULK1 in SH-SY5Y cell autophagy. In target microRNA prediction, we identified miR-595 is a novel ULK1 target miRNA with higher consensus results but miR-4487 is another novel ULK1 target miRNA with lower consensus result. On the contrary, in microarray analyses, we found that miR-4487 is a remarkable as ULK1 target microRNA but miR-595 is not so remarkable. Thus, target microRNA prediction methods should be further improved for suitable to microarray analyses.

Hitherto, some microRNAs have been found to target ULK1-modulated autophagy in different types of diseases. For instance, miR-20a and miR-106b have been reported to negatively regulate autophagy induced by leucine deprivation via suppression of ULK1 expression in C2C12 myoblasts^22^. And, another study has recently reported that the miR-290–295 cluster suppresses autophagic cell death by targeting ULK1 in melanoma cells^23^. Moreover, a mechanistic study has validated that miR-25 inhibition may lead to autophagic cell death by directly increasing ULK1 expression, revealing that miR-25 functions as a novel regulator of autophagy by targeting ULK1^24^. However, to our knowledge, not any report has demonstrated that some microRNAs can target ULK1 in SH-SY5Y cells for potential PD therapeutic purpose. Thus, in our study, we combined *in silico* prediction, microarray analyses and experimental validation to report for the first time that miR-4487 and miR-595 can regulate ULK1-mediated autophagic pathway by targeting ULK1, negatively or positively. These findings not only explore more intricate mechanisms of the two miRNAs (miR-4487 and miR-595) targeting ULK1 in SH-SY5Y cells, but provide new autophagy-related biomarkers of miRNAs for potential PD therapy.

MicroRNA prediction and microarray-based identification were applied for identifying target miRNA of ULK1 in SH-SY5Y cell autophagy. Our data demonstrate that miR-4487 and miR-595 may regulate ULK1-mediated autophagic pathway. Despite increasing evidence linking miRNAs to autophagy in PD, but some important questions remain to be addressed. Although the identification and validation of miRNA targets has greatly improved during the last few years, we know little regarding the cellular and molecular circuits in which they are involved. It also indicates that there is a complicated regulatory network of multiple miRNAs and multiple downstream genes that are important for future studies. The assessment of the potential for miRNAs as biomarkers is only beginning, because greater attention has been paid to the roles of miRNAs in PD. We may next focus on the regulation of miRNAs at different stages of autophagy, or we may consider whether more miRNAs as biomarkers are involved in PD^25^,^26^.

**Figure 5:**
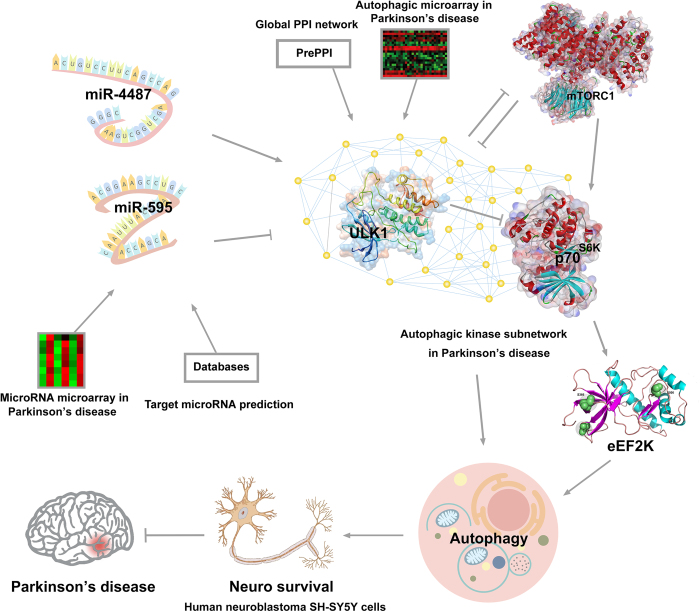
The schematic model of ULK1-mediated autophagic pathway and its target miR-4487 and miR-595 in PD therapy

In conclusion, based upon *in silico* analysis and experimental validation, we found that ULK1 could inhibit p70^S6K^ in starvation-induced autophagy, and further identified that miR-4487 and miR-595 were novel ULK1 target miRNAs (Fig. 5). These findings would shed light on elucidating ULK1 and its target microRNAs as potential targets or biomarkers in the future PD therapy.

## Methods

### Network construction

To construct the basic autophagic kinase network, we download protein interaction data from PrePPI^27^, which is a database of predicted and experimentally determined protein-protein interactions (PPIs) for human. Subsequently, we modified it to the autophagic kinase network based on GO analysis, which was performed using DAVID database (http://david.abcc.ncifcrf.gov/)^28^,^29^. Moreover, we constructed four subnetwork in breast cancer, PD, ageing and type II diabetes according to microarray analysis. According to microarray analysis, we constructed the basic autophagic kinase network. Subsequently, the hub proteins can be based upon the following two standards: Firstly, the degree of each protein in the function-related network was calculated as the number of links that one protein possessed to the other^30^. Hub proteins, identified as their high degrees were extracted based upon the assumption that high-degree protein tended to play a key role in this network. Secondly, we indicate that the hub proteins should connect autophagic kinases thereby making them worth particular focus^31^.

### Target miRNA prediction

Target miRNAs that bound to ULK1 were predicted using miRWalk, a database on predicted and validated microRNA targets^32^. Data from 7 databases: DIANA-mT^33^, miRanda^34^, PICTAR5^35^, miRDB^36^, miRWalk^37^, RNAhybrid^38^ and TargetScan^39^ was used for identifying the high-reliable predicted miRNAs. These results were obtained from overlapping more than 5 databases.

### MiRNA profiling

The SH-SY5Y cells at subconfluency were transfected with ULK1-overexpression plasmid or empty plasmid using LipofectamineTM 2000 Reagent (Invitrogen), following the procedure recommended by the manufacturer. The transfected cells were used for microarray analysis which was carried out following the Phalanx miRNA Hybridization Protocol by Phalanx Biotech Company.

### The significant analysis of microarray (SAM) analysis

The significant analysis of microarray (SAM) method was used to perform the unsupervised calculation. The statistical technique is based on a t-test for finding significant miRNAs in a set of microarray experiments and was proposed. A hierarchical clustering of the differentially expressed miRNAs was performed with Cluster 3.0 (http://bonsai.hgc.jp/~mdehoon/software/cluster/software) version using the average linkage algorithm. The top scoring pair (TSP) algorithm was used to perform the supervised calculation^40^. The basic principle of the k-TSP is to identify miRNA pairs that are oppositely expressed in two classes. All numerical analyses that are presented were performed using Matlab 7.0 (MathWorks Company, Natick, MA, USA).

### Cell culture and reagents

The SH-SY5Y cells were purchased from American Type Culture Collection (ATCC, Manassas, VA, USA). The cells were cultured in DMEM medium supplemented with 10% FBS, 100 μg/ml streptomycin, 100 U/ml penicillin, and 0.03% L-glutamine and maintained at 37 °C with 5% CO_2_ at a humidified atmosphere. All the experiments were performed on logarithmically growing cells. In addition, the SH-SY5Y cells were transfected with ULK1-plasmid for further microRNA microarray. Antibodies against ULK1, p-ULK1 (Ser317), Beclin-1, LC3, p62/SQSTM1, p70^S6K^, p-p70^S6K^ (Thr389), eEF2K, p-eEF2K (Ser366), mTOR, p-mTOR (Ser2448), 4E-BP1, p-4E-BP1 (Ser65), β-actin, RP-conjugated secondary antibodies and siRNA against human ULK1, p70^S6K^ and control siRNA were purchased from Cell Signaling Technology. In addition, rapamycin, 3-MA, miR-4487 mimetic and miR-595 inhibitor were purchased from Sigma (St. Louis, MO, USA).

### Autophagy assay

D-PBS was used to mimicking serum/amino acid (S/AA) starvation in our experiments. Under the condition of starvation-induced autophagy, the SH-SY5Y cells were cultured with 0.05 mM MDC at 37 °C for 60 min. The fluorescence intensity of cells was analyzed by flow cytometry (Becton Dickinson, Franklin Lakes, NJ). Cells were transfected with GFP-LC3 plasmid (kindly provided by Prof. Canhua Huang, Sichuan University) using the Lipofectamine 2000 reagent (Invitrogen) according to the manufacturer’s instructions. The fluorescence of GFP-LC3 was observed under a fluorescence microscope. Also, electron microscopy analysis was performed, to observe the autophagic vacuoles under an electron microscope (Hitachi 7000, Japan).

### SiRNA and miRNA transfection

SH-SY5Y cells were transfected with siRNAs or miR-4487 mimetic and miR-595 inhibitor at 100 nM final concentration using Lipofectamine RNAiMAX reagent (Invitrogen) according to the manufacturer’s instructions. The transfected cells were used for subsequent experiments 48 h later.

### Western blotting

The SH-SY5Y cells were collected, and then western blot analysis was carried out. Briefly, the cell pellets were resuspended with lysis buffer consisting of Hepes 50 mmol/L pH 7.4, Triton-X-100 1%, sodium orthovanada 2 mmol/L, sodium fluoride 100 mmol/ L, edetic acid 1 mmol/ L, PMSF 1 mmol/L, aprotinin (Sigma, MO, USA) 10 mg/L and leupeptin (Sigma) 10 mg/L and lysed at 4 °C for 1 h. After 12,000 × g centrifugation for 15 min, the protein content of supernatant was determined by the Bio-Rad DC protein assay (Bio-Rad Laboratories, Hercules, CA, USA). Equal amounts of the total protein were separated by 10–15% SDS-PAGE and transferred to nitrocellulose membranes, the membranes were soaked in blocking buffer (5% skimmed milk). Proteins were detected using primary antibodies, followed by HRP-conjugated secondary antibody and visualized by using ECL as the HRP substrate.

### Statistical analysis

All the presented data and results were confirmed in at least three independent experiments. The data are expressed as means ± S.D. Statistical comparisons were made by Student’s t-test. *P* < 0.05 was considered statistically significant.

## Additional Information

**How to cite this article**: Chen, Y. *et al*. Identification of ULK1 as a novel biomarker involved in miR-4487 and miR-595 regulation in neuroblastoma SH-SY5Y cell autophagy. *Sci. Rep*. **5**, 11035; doi: 10.1038/srep11035 (2015).

## Supplementary Material

Supplementary Information
