# Supplementary material for: Identification of ULK1 as a novel biomarker involved in miR-4487 and miR-595 regulation in neuroblastoma SH-SY5Y cell autophagy
Source: Sci Rep. 2015 Jul 17;5:11035. doi: 10.1038/srep11035 (PMC4505320; doi:10.1038/srep11035)
Supplement: Supplementary Information [file srep11035-s1.pdf]

Supplementary information

Identification of ULK1 as a novel biomarker involved in miR-4487 and miR-595 regulation in neuroblastoma SH-SY5Y cell autophagy

Yi Chen[1#], Shuya Wang[1, 2#], Lan Zhang[1, 3#], Tao Xie[1], Sicheng Song[1], Jian Huang[3], Yonghui Zhang [1, 4], Liang Ouyang[1*] & Bo Liu[1*]

[1]Department of Gastrointestinal Surgery, State Key Laboratory of Biotherapy, Collaborative Innovation Center of Biotherapy, West China Hospital, Sichuan University, Chengdu 610041, China, [2]Northwestern University, Feinberg School of Medicine, 303 East Chicago Avenue, Chicago, Illinois 60611, USA, [3]School of Traditional Chinese Materia Medica, Shenyang Pharmaceutical University, Shenyang, 110016, China, [4]Collaborative Innovation Center for Biotherapy, Department of Pharmacology & Pharmaceutical Sciences, School of Medicine, Tsinghua University, Beijing 100084, China

Running title: ULK1 and its target miRNAs in autophagy

[#]These authors contributed equally to this work. [*]Corresponding author. Tel/Fax: 86-28-85164063. E-mail addresses: liubo2400@scu.edu.cn (Bo Liu); ouyangliang@scu.edu.cn (Liang Ouyang).

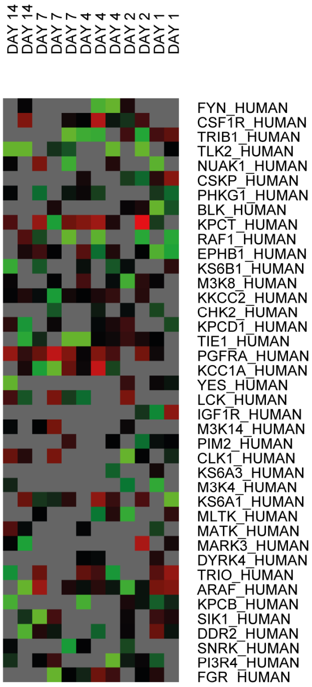

A. Autophagic microarray in breast cancer (GSE22386)

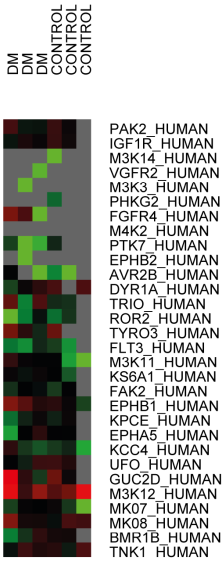

C. Autophagic microaaray in type II diabetes (GSE21340)

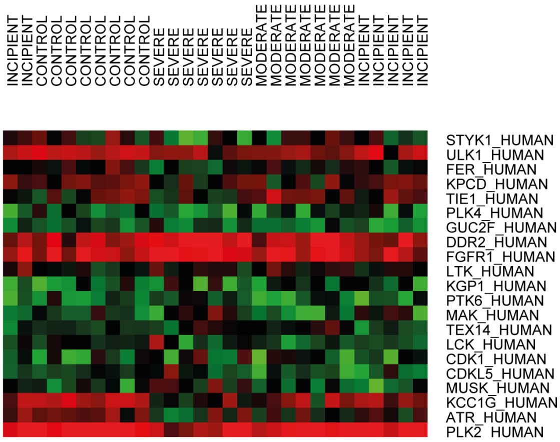

B. Autophagic microarray in Parkinson's disease (GSE28894)

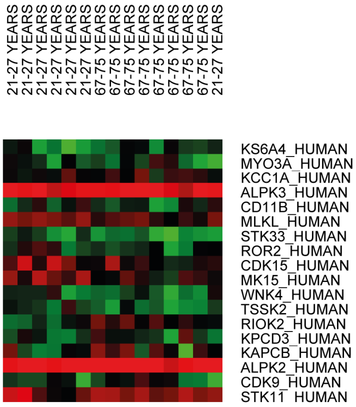

D. Autophagic microarray in ageing (GSE236)

Figure S1 | Different types of microarray evidence in human diseases

(A) Breast cancer; (B) Parkinson's disease; (C)Type II diabetes; (D) Ageing.

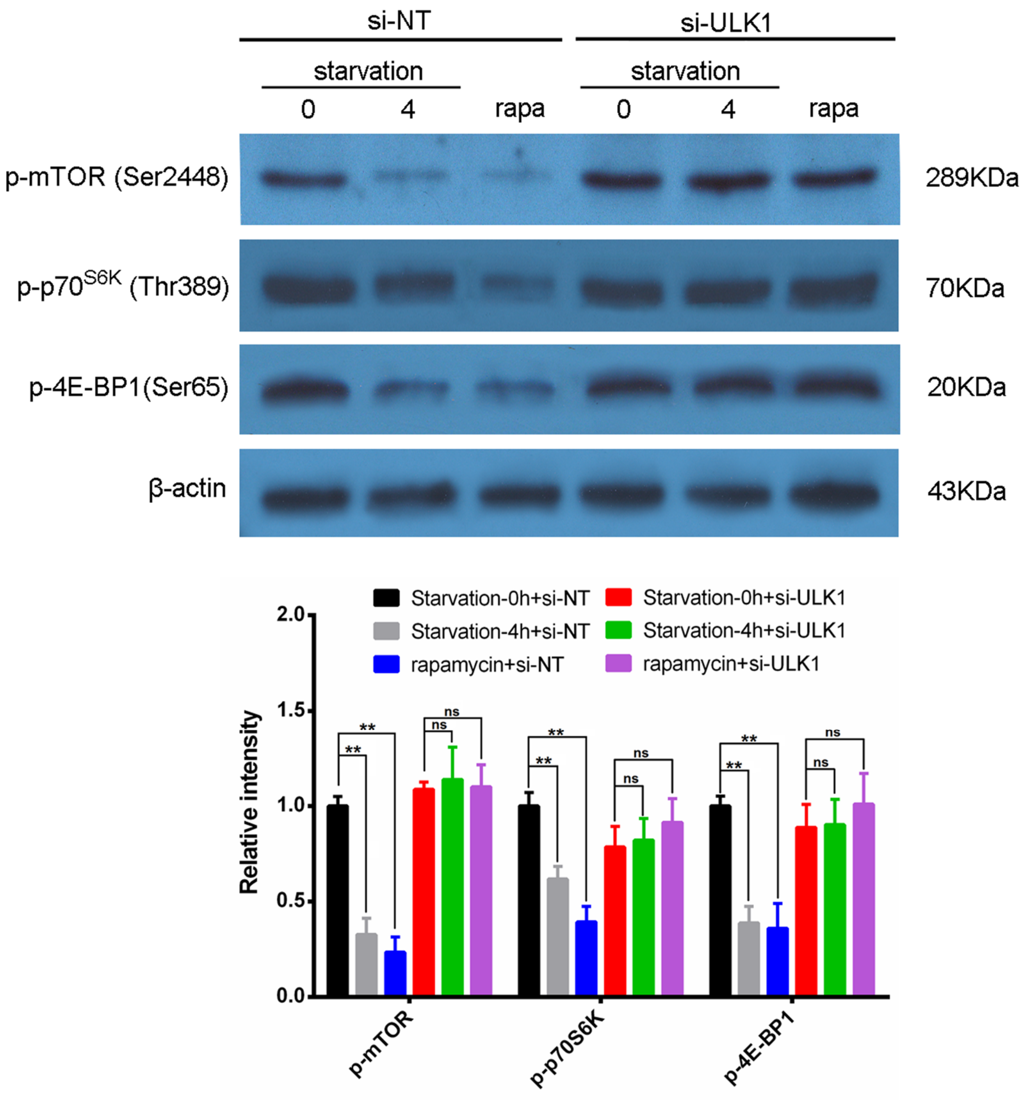

Figure S2 | ULK1 may regulate p70^{S6K} via mTOR

The SH-SY5Y cells were transfected with siRNAs for 48 h, followed by starvation for 0, 4 h, or treatment of rapamycin (1μM) for 4h, then subjected to immunoblot, β-actin was used as a loading control, the data are representative of 3 independent experiments. *P < 0.05; **P < 0.01.
